# Seroprevalence of hepatitis B virus among antenatal clinic attendees in Gamawa Local Government Area, Bauchi State, Nigeria

**DOI:** 10.1186/s12879-020-4863-9

**Published:** 2020-03-06

**Authors:** Garba Umar Mustapha, Abdulrasul Ibrahim, Muhammad Shakir Balogun, Chukwuma David Umeokonkwo, Aisha Indo Mamman

**Affiliations:** 1 Nigeria Field Epidemiology and Laboratory Training Programme, Abuja, Nigeria; 2 Department of Community Medicine, Ahmadu Bello University, Zaria, Kaduna State Nigeria; 3 State Primary Health Care Development Agency, Bauchi, Bauchi State Nigeria; 4 Department of Medical Microbiology, Ahmadu Bello University Teaching Hospital, Zaria, Kaduna State Nigeria; 5 Department of Community Medicine, Alex Ekwueme Federal University Teaching Hospital, Abakaliki, Ebonyi State Nigeria; 6 Department of Haematology, Ahmadu Bello University Teaching Hospital, Zaria, Kaduna State Nigeria

**Keywords:** Hepatitis B virus, Hepatitis B surface antigen, Prevalence, Pregnancy, Health facilities, Hepatitis B

## Abstract

**Background:**

Hepatitis B virus disease is a potentially life-threatening liver infection and a major global health problem. It causes chronic infection and puts people at high risk of death from cirrhosis and liver cancer. WHO estimated 257 million people are living with hepatitis B virus (HBV) infection and in 2015 alone HBV resulted in to 887,000 deaths globally. We determined the prevalence and associated factors of hepatitis B virus infection among Antenatal Care (ANC) attendees in Gamawa Local Government Area, Bauchi State.

**Methods:**

We conducted a descriptive cross-sectional, health facility-based study between March and April 2018. We used systematic random sampling technique to recruit 210 pregnant women aged 15–49 years. With a structured questionnaire, we interviewed the respondents and collected blood sample to test for hepatitis B surface antigen. We calculated frequencies, means, proportions, and tested for associations using Epi Info 7.2 and Microsoft Excel.

**Results:**

The mean age of respondents was 24.5 ± 6.0 years; 112 (53%) of whom were younger than 25 years. All were married, 183 (87%) had no formal education and up to 190 (90%) were employed. Overall, 14 (6.7%) tested positive for HBsAg; women aged ≥35 years had the highest prevalence (10%). None with tertiary education tested positive and women married before 18 years had 13 (6.2%) prevalence.

**Conclusions:**

The prevalence of HBsAg among pregnant women in Gamawa LGA was 6.7% which is quite lower than the national prevalence reported. We recommended improved surveillance of HBV infection and screening of women attending ANC.

## Background

Hepatitis B virus disease is a potentially life-threatening liver infection and a major global health problem. Hepatitis B infection is associated with the risk of death arising from cirrhosis, liver and non-liver cancers [1]. Transmission of Hepatitis B virus (HBV) results from exposure to contaminated blood or body fluids, unprotected sexual contact with an infected person, blood transfusion, use of contaminated needles, syringes, and sharps as well as vertical transmission from mother to child. There is risk of HBV transmission to the newborn delivered by a hepatitis B surface antigen (HBsAg)-positive woman. This risk is amplified with positivity of hepatitis B envelope antigen (HBeAg) [2].

Globally, WHO estimates that in 2015, 257 million persons, or 3.5% of the population, were living with chronic HBV infection. The African and Western Pacific regions accounted for 68% of those infected. In Nigeria, the National Program on Immunization (NPI) which targets the infants, started in 1998. Hepatitis B vaccine coverage for infants were 50.6% for first dose, 45.6% second dose and 38.2% for third dose [3]. In Bauchi State the vaccination coverage ranges from 25.4% for first dose, 18.9% second dose and 12.5% for third dose [3]. The third dose of Pentavalent vaccine coverage among children has increased from 12.5% in 2013 to 32.1% in 2018 [3, 4].

Several authors have reported on the prevalence of HBV among sub-populations worldwide with estimates varying depending on population studied and methods used. Reported prevalence of HBsAg among pregnant women varies from one region to another. The prevalence of chronic HBV infection was estimated to be 3.5% among women of reproductive age globally [5]. In African countries the prevalence ranges within 6–25% [5]. A national survey of hepatitis B in Nigeria showed prevalence of 12.2% among the general population [6], a systematic review of hepatitis B infection among pregnant women in Nigeria found a prevalence of 14.1% [7]. HBsAg prevalence documented among pregnant women in Bayara, Bauchi State was 17.2% [8].

Many authors report on the prevalence of HBV among sub-populations in Nigeria in secondary and tertiary healthcare settings and usually in urban areas, with estimates varying depending on population studied and methods used. However, there is dearth of reliable data on prevalence of HBV infection among pregnant mothers at primary health care level especially remote areas. Also, despite Nigeria being categorized among countries where HBV infection is highly endemic [9], Gamawa LGA reported zero case of HBV infection to the state surveillance system for five consecutive years (2011–2015). We therefore aimed to determine the prevalence of HBV infection among pregnant mothers attending ANC at PHCs of Gamawa LGA.

## Methods

### Study design and setting

We conducted a cross-sectional study among women receiving antenatal care (ANC) in Primary Health Centres (PHCs) of Gamawa LGA, Bauchi State between March and April 2018. Gamawa LGA has an estimated projected population of 427,761 (2018) [10]. It is a rural LGA and farming is the predominant occupation of the people. The LGA has 18 political wards and 62 PHCs out of which 24 offered ANC services. Among the PHCs that offered ANC, the services were offered once weekly in most of the health facilities and average ANC attendance was 30 clients per facility per day.

### Sample size determination

We calculated a sample size of 210 using the formula for cross sectional studies [11] based on the documented HBsAg prevalence of 14.1% among pregnant women in Nigeria [7] at 95% confidence interval (CI), 0.05 precision and 10% non-response rate.

### Sampling technique

We employed multistage sampling method to recruit 210 pregnant women. At the first stage, we recruited eight health facilities (HFs) from the list of 24 HFs that provide ANC using balloting method of simple random sampling. At the second stage, we recruited clients attending ANC in the selected HFs using systematic random sampling. We planned to collect the sample over 8 weeks. Using the average daily attendance to ANC in the selected hospitals, we estimate a sampling interval of 10. The first attendee is sampled by balloting and subsequently every 10th attendee was approached for the study till the sample size was achieved. After explaining the purpose of the study, written informed consent was obtained, the questionnaire was administered, and one millilitre of blood sample was collected through an aseptic venipuncture for HBV serology.

### Study instrument and data collection

The participants were interviewed by the investigator and research assistants using a semi-structured questionnaire to obtain information on socio-demographic characteristics, history of vaccination, blood transfusion, surgery, tonsillectomy, dental procedure, sharing of needles, and history of cupping, scarification and episiotomy. The questionnaires were both in English and Hausa. After this one millilitre of blood was collected aseptically for hepatitis B serology. We excluded ANC attendees that were not residing in Gamawa LGA.

### Laboratory method

HBsAg was assayed using an enzyme-linked immunosorbent assay (ELISA) kit produced by LabACON^R^ (Hangzhou Biotest Biotech Co., Ltd., China) which has sensitivity and specificity of 99.9 and 99.0% respectively. The 99.9 and 99.0% for sensitivity and specificity are company declared figures which are similar to other commercial figures. The kit has in-built controls. The manufacturer’s instruction were closely followed. The results were reported as positive or negative.

### Statistical analyses

Data analysis was performed using Microsoft Excel and Epi-Info 7.2. We estimated the proportion of pregnant women positive to HBsAg, frequency and proportion of the various sociodemographic and clinical characteristics. We examined the relationship between the HBsAg status and the sociodemographic and clinical characteristics using the chi square statistics at the 95% confidence interval. All the women contacted for the study have agreed and participated in the Study, there were no issues with test method and collection of data.

## Result

A total of 210 pregnant women were enrolled in the study. The mean age was 24.5 ± 6.0 years, median age was 24 years (interquartile range 20–28 years). All were married, 87% had no formal education and up to 91% were employed. Overall, 6.7% (3.8–10.7%) tested positive for HBsAg, those older than 35 years had the highest prevalence of 10.5% (1.8–30.6%, Table 1). None was positive among those with tertiary education, and women married before 18 years had 6.5% (3.5–10.7%) prevalence. There was no statistically significant difference between the proportions of HBsAg positives and negatives with respect to history of vaccination, blood transfusion, surgery, tonsillectomy, dental procedure, sharing of needles, history of cupping, scarification and episiotomy. There was also no statistically significant difference in the proportion of HBsAg positives and negatives with respect to age, level of education, employment status, husband’s occupation, parity, age at the time of first marriage and number of marital units. (Table 2).

**Table 1:** Socio-demographic characteristics and prevalence of HBsAg among women attending ANC in PHCs of Gamawa LGA, Bauchi State, Nigeria (*N* = 210)

Variable	HBsAg + ve	HBsAg-ve	Prevalence (95% CI)	Total
Overall prevalence	14	196	6.7 (3.8–10.7)	210
Age group (years)				
15–24	7	105	6.3 (2.8–12.0)	112
25–34	5	74	6.3 (2.4–13.5)	79
≥35	2	17	10.5 (1.8–30.6)	19
Educational Level				
No formal/Qur’anic	11	172	6.0 (3.2–10.2)	183
Primary	1	15	6.2 (0.3–27.2)	16
Secondary	2	8	20.0 (3.5–52.0)	10
Tertiary	0	1	0.0 (0–95)	1
Occupation				
Farmer	6	83	6.7 (2.8–13.5)	89
Trader	4	57	6.6 (2.1–15.1)	61
Tailor	2	25	7.4 (1.3–22.4)	27
Housewife	1	19	5.0 (0.2–22.3)	20
Others	1	12	7.8 (0.4–32.5)	13
Employment Status				
Employed	10	163	5.8 (3.0–10.1)	173
Unemployed	4	47	7.8 (2.5–17.8)	51
Parity				
Primiparous	6	56	9.7 (4.0–19.0)	62
Multiparous	8	140	5.4 (2.5–10.0)	148
Age at First Marriage				
< 18	12	174	6.5 (3.5–10.7)	186
≥18	2	22	8.3 (1.4–24.9)	24
Number of sexual partners				
1	10	148	6.3 (3.3–11.0)	158
> 1	4	48	7.7 (2.5–17.5)	52

**Table 2:** Factors associated with HBV infection among women attending ANC in PHCs of Gamawa LGA, Bauchi State, Nigeria (*N* = 210)

Risk factor	Hepatitis B status		OR (95% CI)
	Positive n (%)	Negatives n (%)	
Age (Years)			
≤30	12 (6.5)	174 (93.5)	0.8 (0.16–3.61)
> 30	2 (8.3)	22 (91.7)	
Education			
≤ Primary	12 (6.0)	187 (94.0)	0.3 (0.06–2.17)
> Primary	2 (18.2)	9 (81.8)	
Employment Status			
Employed	13 (6.7)	181 (93.3)	1.1 (0.13–8.81)
Unemployed	1 (6.2)	15 (93.8)	
Parity			
Primiparous	6 (9.7)	56 (90.3)	1.9 (0.62–5.65)
Multiparous	8 (5.4)	140 (94.6)	
Age at first marriage			
< 18	12 (6.5)	174 (93.5)	0.8 (0.16–3.61)
18 and above	2 (8.3)	22 (91.7)	
Number of sexual partners			
1	10 (6.3)	148 (93.7)	0.8 (0.24–2.70)
> 1	4 (7.7)	48 (92.3)	
History of blood transfusion			
Yes	2 (9.5)	19 (90.5)	1.6 (0.32–7.46)
No	12 (6.3)	177 (93.7)	
History of surgery			
Yes	1 (25.0)	3 (75.0)	4.9 (0.48–50.96)
No	13 (6.3)	193 (93.7)	
History of Uvulectomy			
Yes	0 (0.0)	18 (100.0)	
No	14 (7.3)	178 (92.7)	
Local dental procedure			
Yes	0 (0.0)	4 (100.0)	
No	14 (6.8)	192 (93.2)	
Sharing of needles			
Yes	0 (0.0)	4 (100.0)	
No	14 (6.8)	192 (93.2)	
Cupping			
Yes	2 (4.1)	47 (95.9)	0.5 (0.11–2.45)
No	12 (7.5)	149 (92.5)	
Scarification			
Yes	4 (8.5)	43 (91.5)	1.4 (0.43–4.76)
No	10 (6.1)	153 (93.9)	
Vaccination			
Yes	0 (0.0)	9 (100.0)	
No	14 (7.0)	187 (93.0)	

## Discussion

HBV infection affecting pregnant women may result in maternal morbidity and mortality. It could also result in chronic infection in the newborn. Prevalence of hepatitis B infection varies in different parts of the world. Even within the same country there are regional and population specific variations. Immunosuppression in pregnancy is of clinical and epidemiological relevance with regards to hepatitis B viral infection [12]. In this study the overall prevalence of HBsAg among women attending antenatal care services in PHCs in Gamawa LGA was 6.7% indicating an intermediate endemicity. This contrasts with reports by Colin *et al* [9] that classified Nigeria in the high endemicity group. The finding from our study supports the fact that the prevalence of HBV infection varies widely based on location and subpopulations. The implication of this finding is that pregnant women, even in rural communities, are at high risk of HBV infection, hence the need for HBV screening during antenatal care services. The result from our study is similar to that from a study done by Oluboyo *et al* [12] and is in agreement with prevalence of 6–25% in the WHO African region. Suffice to note that similar results by all study groups may be linked to identical WHO approved test kits.

The prevalence is lower than a similar study conducted in Bayara hospital, Bauchi State where a prevalence of 17.2% was documented [8]. The difference could be due to the fact that it was conducted in secondary facility situated in urban area. A prevalence of 8.2% was reported also from north eastern Nigeria in FMC Yola, Adamawa State [13]. In the North-Central geopolitical zone, a prevalence of 12.3% was reported from Minna Niger State by Ndams *et al* [14] and 11.0% was reported in Makurdi, Benue State by Mbaawuaga et al. [15]

In the south-western part of the country, a prevalence of 16.5% was reported in Osogbo, Osun State by Kolawole *et al* [16] and 8.3% in Ibadan by Chinenye et al. [17] Southern states reported a prevalence of 12.5% in Edo State by Ugbebor et al. [18] It is also lower than the 9.6–18.6% observed by Musa et al [7] in a country wide systematic review. Furthermore, it is lower than the 9.7% found among pregnant women attending ANC in Bue’a Health District [19], 10.2% found by Noubiap *et al* [20], 20.4% found by Ducancelle [21] *et al* in Cameroon. It’s also lower than the 9.5% found in Ghana by Ephraim *et al* [22] and 9.3% found in Kenya by Okoth *et al* [23]. The differences in the reported seroprevalence rates of HBV among pregnant women may be due to variation in the geographical location, socio-cultural practices, study design, level of care for the study facility, sample size and test methods employed. Most of the studies quoted above were carried out at secondary and tertiary level of care located mostly in urban or semi urban populations but this study was carried out at primary health care setting in rural communities. The lower prevalence of HBV compared to the previous Nigerian studies may be due to increased awareness about the disease [24].

However, the prevalence from our study is higher than the 4.3% found among pregnant women in Zaria, north-western-Nigeria, 5.3% in Yenagoa, Bayelsa State; 1.6% found in Iran [25]; 3.9% found in pregnant women attending ANC in Muhimbili national hospital Tanzania [26] and 5.6% in Sudan [27]. It was similar to a prevalence of 6.8% reported in Ekiti State, southwestern Nigeria [28], and 6.6% in Cross River State [29] southsouth Nigeria. The differences may reflect variations in overall health care status of the study locations. The cost of screening could have accounted for lack of surveillance reports from Gamawa. The cost of screening for HBV in the community was $1.39 per client. The amount is considered high for the average person in the community due to high level of poverty in the rural community. Since they are apparently healthy, it becomes difficult to convince the women to part with the money that would have rather been used for food. Making these screenings free during antenatal visits would help a lot in ensuring that every woman knows her status and take steps to improve her health and protect her unborn baby. PHC facilities hence do not routinely screened for HBV infection.

## Conclusion

The prevalence of HBsAg among pregnant women in Gamawa LGA was 6.7% which is much lower than the national prevalence of 14.1% among ANC attendees. Women older than 35 years had the highest prevalence of 10% and of the documented associated factors studied, none was found to be associated with HBV infection. We recommended free HBV screening for women attending ANC and more awareness campaigns on hepatitis B virus infection and its consequences especially among pregnant women.

## Data Availability

The datasets used and/or analysed during this study are available from the corresponding author on reasonable request.

## Abbreviations

ANC: Ante Natal Care
CI: Confidence Interval
ELISA: Enzyme Linked Immunosorbent Assay
FMC: Federal Medical Centre
HBeAg: Hepatitis B envelope antigen
HBsAg: Hepatitis B surface antigen
HBV: Hepatitis B Virus
HF: Health Facility
LGA: Local Government Area
NPI: National Programme on Immunization
PHC: Primary Health Centres
WHO: World Health Organisation
